# Decomposing Juggling Skill into Sequencing, Prediction, and Accuracy: A Computational Model with Low-Gravity VR Training

**DOI:** 10.3390/s26010294

**Published:** 2026-01-02

**Authors:** Wanhee Cho, Makoto Kobayashi, Hiroyuki Kambara, Hirokazu Tanaka, Takahiro Kagawa, Makoto Sato, Hyeonseok Kim, Makoto Miyakoshi, Scott Makeig, John Rehner Iversen, Natsue Yoshimura

**Affiliations:** 1 Department of Information and Communications Engineering, Institute of Science Tokyo (Suzukakedai Campus), 4259 Nagatsuta-cho, Midori-ku, Yokohama 226-8501, Kanagawa, Japan; wani.cho@brain.comp.isct.ac.jp (W.C.); mkt.sato@gmail.com (M.S.); 2 Information Technology Course, Faculty of Engineering, Tokyo Polytechnic University (Atsugi Campus), 5-45-1 Iiyamaminami, Atsugi 243-0297, Kanagawa, Japan; h.kambara@eng.t-kougei.ac.jp; 3 Tokyo City University (Setagaya Campus), 1-28-1 Tamazutsumi, Setagaya-ku, Tokyo 158-8557, Japan; htanaka@tcu.ac.jp; 4 Faculty of Engineering, Department of Mechanical Engineering, Aichi Institute of Technology (Yakusa Campus), 1247 Yachigusa, Yakusa-cho, Toyota 470-0392, Aichi, Japan; t_kagawa@aitech.ac.jp; 5 Cincinnati Children’s Hospital Medical Center (Burnet Campus), 3333 Burnet Avenue, Cincinnati, OH 45229-3026, USAMakoto.Miyakoshi@cchmc.org (M.M.); 6 Swartz Center for Computational Neuroscience (SCCN), Institute for Neural Computation, University of California San Diego, 9500 Gilman Drive #0559, La Jolla, CA 92093-0559, USA; smakeig@ucsd.edu; 7 Department of Psychology, Neuroscience & Behaviour, McMaster University, 1280 Main Street West, Hamilton, ON L8S 4L8, Canada; jiversen@mcmaster.ca

**Keywords:** 3-ball juggling, computational model, computer vision, motion capture, multimodal evaluation system

## Abstract

Juggling is a complex motor skill that requires multiple sub-skills and cannot be mastered without extensive practice. Although prior studies have quantified performance differences between novice and expert jugglers, none have attempted to quantitatively decompose these components or model their contribution to juggling performance. This longitudinal study presents a multimodal evaluation system that integrates computer vision, motion capture, and biosensing to quantify three key elements of juggling ability: Sequencing, Prediction, and Accuracy. Twenty beginners completed a 10-day, three-ball juggling experiment combining visuo-haptic virtual reality (VR) and real-world practice, with half training in reduced gravity, previously shown to enhance early-stage motor learning. The fitted Gamma-Log generalized linear model (GLM) indicated that Sequencing is the dominant factor of early skill acquisition, followed by Prediction and Accuracy. This study provides the first computational decomposition of juggling, demonstrates how multiple elements jointly contribute to performance, and results in a principled approach to characterizing motor learning in complex real-world tasks.

## 1. Introduction

The acquisition of motor skills refers to long-lasting improvements in movement ability through practice, which are shown through their retention and transfer to new situations [1]. Motor learning occurs on multiple time scales: rapid gains can occur within a single practice session, while slower consolidation occurs over days or weeks [2]. Classic stage models describe skill acquisition as a progression from cognitively demanding, error-prone performance to more automatic, stable execution [3,4]. In parallel, dynamical systems and computational motor control theories emphasize how coordinated movement patterns emerge and how internal models and optimal feedback control are used to reduce variability and improve efficiency [5,6,7]. Together, these views suggest that complex skills are not supported by a single factor but by multiple interacting sub-processes (e.g., timing, prediction, spatial precision) that may change at different rates during learning. Although motor learning has been widely studied at the behavioral and training levels, building computational models that map these theoretical ideas onto concrete, measurable components remains challenging because human movement involves many redundant degrees of freedom [5], individuals differ in their strategies and constraints, and performance is strongly affected by task context [7]. In general, these factors make it difficult to isolate the contribution of specific elements and to connect them to overall skill.

Three-ball cascade juggling provides a useful model system for studying complex motor learning. Performance can be quantified in a relatively objective way, for example, by measuring how long a participant can maintain the pattern or by counting successful throws and catches [8]. At the same time, juggling is a complex skill because it requires several physical and cognitive processes to be coordinated simultaneously. Learners must perform consistent throws (e.g., stable speed and release angle), track multiple balls visually, and maintain rhythmic timing for continuous catch-throw cycles [9]. Because these components must be tuned together, most people cannot acquire juggling skill without sufficient practice.

For these reasons, juggling has been used in many physiology and neuroscience studies as a model of complex motor skill acquisition. Training-related changes in white matter architecture, including regions near the intraparietal sulcus, have been reported using diffusion tensor imaging (DTI) [10], and increases in gray matter volume in visual-processing areas have been observed using voxel-based morphometry (VBM) [11]. Behavioral studies also show that expert jugglers use more efficient gaze strategies, with tighter gaze–ball coupling and reduced horizontal gaze shifts, consistent with predictive perception–action control [12]. In addition, spatiotemporal variability in cascade juggling shows regular patterns rather than random noise, so rhythm stability and coordination can be quantified [13].

However, most prior studies did not systematically decompose juggling into multiple interacting motor and cognitive elements. Some work has begun to extract lower-dimensional, interpretable components. Post et al. used principal component analysis (PCA) to reveal low-dimensional coordination structures underlying three-ball cascade juggling [14]. Müller and Sternad showed that skill gains reflect structured changes in execution variability, namely noise reduction, tolerance exploitation, and covariation (N-T-C), rather than a simple decrease in variance [15]. Ramsay et al. applied functional data analysis (FDA) to juggling trajectories and introduced landmark and continuous registration methods to separate phase and amplitude, allowing cycle-wise alignment across participants and sessions [16]. Finally, Yamamoto and colleagues found that learning does not change smoothly. Instead, it advances in stages constrained by the task, with stable dwell-time ratio levels (related to the Farey sequence), and adaptability differs between coordination patterns, which indicates pattern-specific learning dynamics [17,18]. Despite these advances, two key gaps remain in a computational explanation of how multiple elements work together to determine overall performance. First, most studies focus on a single facet (e.g., PCA [14], FDA [16], or N-T-C [15]), so there is no integrated framework that can estimate multiple candidate elements at the same time. Second, decomposed measures are rarely linked to a single predictive model that explains overall juggling performance with formal validation.

Outside the juggling domain, recent multimodal studies in transportation and human–machine interactions show how synchronized behavioral and sensor data can be used to estimate latent cognitive or motor states, for example, by combining gaze and control inputs to model pilots’ situation awareness [19] and integrating inertial, physiological, and environmental signals for robust human activity recognition [20]. More general data-driven frameworks for motor learning also emphasize the fusion of kinematic, physiological, and contextual information to describe how complex skills change over time [21]. In rehabilitation engineering, adaptive exoskeleton controllers based on admittance models adjust assistance according to the user’s evolving motor state [19]. These studies highlight the value of multimodal sensing and component-level descriptions of motor ability.

Building on these insights, we develop a component-based computational model of early juggling learning. In this model, juggling skill is described by three complementary facets suggested by motor control theory: spatiotemporal coordination (Sequencing), predictive state estimation (Prediction), and spatial precision (Accuracy). Sequencing (*S*) captures the rhythmic, phase-locked organization of throws and catches across cycles, corresponding to the stable coordination patterns and movement synergies in dynamical system frameworks [4,5]. Prediction (*P*) reflects the ability to estimate ball states during visual occlusion and to perform appropriate catch actions based on internal models of object dynamics, in line with optimal feedback control and internal model theories [6,7]. Accuracy (*A*) quantifies the spatial consistency of second-catch locations in a hand-centered frame, indexing endpoint precision and control of execution variability, as emphasized in variability-based accounts of skill [15]. These three sub-skills form a low-dimensional, interpretable set that can be directly derived from our kinematic and occlusion data and linked to overall juggling performance in a single computational model.

We also considered other possible decompositions. One alternative is to treat juggling as mainly feedforward once the cascade becomes stable, where performance could be explained mostly by Sequencing and Accuracy without an explicit Prediction component. However, in early learning, the ball trajectory is not consistent across cycles, so novices likely rely on prediction-based state estimation to support rapid online corrections. This view aligns with optimal feedback control and internal model accounts, which emphasize predicting movement outcomes and using feedback to reduce the impact of motor variability [6,7,22]. For this reason, we retain Prediction as a distinct component and probe it using the visual-occlusion catching task.

Also, among these components, Sequencing reflects the ordered and rhythmic structure of the cascade, which is often the main difficulty when beginners start learning. Based on this idea, our visuo-haptic VR training system was designed to slow down ball movement by reducing gravity so that novices can perceive and practice the juggling rhythm with less time pressure. Because the only experimental intervention between groups was this low-gravity slow-tempo training, we also explore whether group differences appear mainly in Sequencing as a behavioral check of the intended training effect.

**Figure 1:**
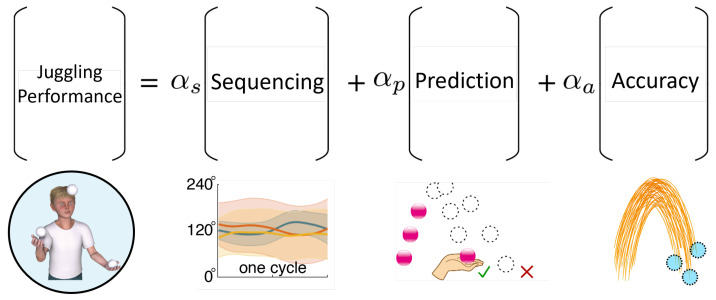
Conceptual decomposition of juggling skill into three components. Juggling performance (Perf) is modeled as a weighted combination of Sequencing (*S*), Prediction (*P*), and Accuracy (*A*), with coefficients $\alpha_{S}$, $\alpha_{P}$, and $\alpha_{A}$. Sequencing represents spatiotemporal coordination among balls across cycles, illustrated schematically by phase relations within a cycle. In the Prediction panel, √ and × indicate successful and failed catch attempts, respectively, and dashed circles represent example ball positions of a visually occluded ball. In the Accuracy panel, circles illustrate example catch locations used to quantify the spatial consistency of catch locations (as defined in Methods).

Based on this framework, we tested three hypotheses regarding the computational structure of juggling skill:

**Hypothesis** **1** (H1)**.** *Juggling can be decomposed into three latent components: Sequencing (S), Prediction (P), and Accuracy (A) (Figure 1). Following our previous definitions [23], S represents spatiotemporal coordination among balls across cycles, P represents the ability to predict the landing position of a visually occluded ball, and A represents the spatial consistency of catch locations.*

**Hypothesis** **2** (H2)**.** *Day-level juggling performance (Perf) can be modeled as a function of S, P, and A, i.e., Perf=f(S,P,A), with independent and complementary contributions from each component.*

**Hypothesis** **3** (H3)**.** *During early learning, S, P, and A do not increase at the same rate, but show lead–lag relationships across days (asymmetric development). Because Sequencing is a main bottleneck for beginners, we expect slow-tempo low-gravity VR training to mainly promote early Sequencing gains.*

The contribution of this study is four-fold:

1. Building on our previous VR study, we report, to our knowledge, the first 10-day juggling training multimodal evaluation system that combines computer vision (ball and hand kinematics), motion capture (3D wrist and shoulder trajectories), and biosignals (EEG and EMG), all synchronized on a common time base.
2. We develop a fully automatic analysis pipeline that replaces manual video scoring and extracts trial duration, number of successful catches, and ball trajectories at scale from the multimodal recordings.
3. Using these extracted measures, we innovatively define three components—Sequencing, Prediction, and Accuracy—and connect them in a computational model that jointly explains day-level juggling performance.
4. Leveraging the longitudinal design with repeated measurements in novices, we quantify how these three components change across days and demonstrate that they follow distinct learning rates during early acquisition.

The remainder of this paper is structured as follows: Section 2 outlines the experiment setup, including the research hypotheses; experimental design; sensors and data collection; the construction of Sequencing, Prediction, and Accuracy indices; and statistical modeling. Section 3 presents the main findings, followed by further interpretation and discussion in Section 4, together with limitations and future works. Section 5 concludes this study.

## 2. Materials and Methods

This study used the same ten-day juggling experiment as our previous work on VR-based motor learning, which introduced the behavioral effects of VR training on real-world juggling. We introduced different gravity-level settings in the VR training in two groups: a progressive low-to-normal gravity group (hereafter, Low-g group) or a constant normal-gravity group (hereafter, Normal-g group). This paper focuses on building three skill indices, Sequencing (S), Prediction (P), and Accuracy (A), from kinematic data and modeling day-level juggling performance as a function of these indices. Detailed descriptions of the visuo-haptic VR setup, gravity intervention, and biosensor recordings (EEG, EMG, and functional magnetic resonance imaging (fMRI)) are provided in the work of Cho et al. [23].

### 2.1. Participants

Twenty healthy adults with no prior juggling experience joined the experiment (14 males, 6 females; age 24.1±3.5 years). Participants were randomly assigned, with matched sex ratios, to either a Low-g group (n = 10; 7 males, 3 females; age 24.8±4.1 years) or a Normal-g group (n = 10; 7 males, 3 females; age 23.4±2.8 years). All participants provided written informed consent. The study protocol was approved by the ethics committee of the Tokyo Institute of Technology (currently, Institute of Science Tokyo) (approval no. 2020107).

### 2.2. Experimental Design

Each participant completed 10 consecutive days of training. On each day, the following four sessions were performed in a fixed order:

Session 1: Real-world three-ball juggling (pre).
Session 2: VR three-ball juggling.
Session 3: Visual-occlusion juggling with shutter glasses.
Session 4: Real-world three-ball juggling (post).

**Figure 2:**
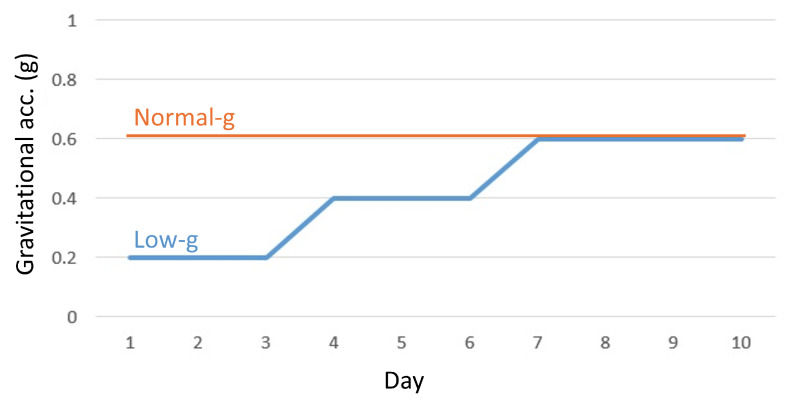
Daily gravity acceleration in the VR system. The blue line shows the slightly increasing gravity of the Low-g group, while the orange line shows the constant gravity of the Normal-g group. Adapted from [23], with modifications ©2025 IEEE.

The visuo-haptic VR training in Session 2 includes low-gravity intervention and group allocation (progressive Low-to-Normal gravity vs. constant gravity), as described in detail by Cho et al. [23]. The training protocol used a slow-tempo, low-gravity visuo-haptic VR system; we hope that by reducing gravitational acceleration and varying the balls’ flight time, the learning of cascade rhythms may be promoted, potentially improving the development of Sequencing during early practice. Briefly, the Low-g group practiced with progressively increasing gravity (0.2 g, 0.4 g, and 0.6 g on Days 1–3, 4–6, and 7–10, respectively), whereas the Normal-g group trained at a constant 0.6 g, as shown in Figure 2. This study focuses on kinematic indices and their relationship to juggling performance, while group-wise patterns are presented descriptively to explore the behavioral trends. The full experiment was designed as a multimodal platform combining kinematic recordings, VR logs, EEG, EMG, and pre-/post-training fMRI.

### 2.3. Sensors and Data Collection

#### 2.3.1. Overview of Sensors

The experiment collected multimodal data from kinematic, VR, and neurophysiological systems. All devices were time-synchronized using Lab Streaming Layer (LSL; liblsl v1.13.0) [24]. In the present analysis, we only used the kinematic streams from motion capture, RGB-D video, and shutter timing to compute the Sequencing (S), Prediction (P), and Accuracy (A) indices. The remaining modalities are listed briefly to describe the overall study design.

- Optical motion capture (used for P, A): An OptiTrack (NaturalPoint Inc., Corvallis, OR, USA) system recorded 3D positions of four reflective markers placed on both shoulders and both wrists at 200 Hz.
- RGB-D camera (used for S, P, A): A front-facing Intel RealSense D455 (Intel Corporation, Santa Clara, CA, USA) recorded color video and depth at 60 Hz during real-world juggling and the occlusion task, capturing ball trajectories and hand movements (librealsense2 v2.49.0).
- Shutter-glasses trigger (used for P): signals indicating the opening and closing of liquid-crystal shutter glasses (PLATO Visual Occlusion Spectacles; Translucent Technologies Inc., Toronto, ON, Canada [25]) were recorded as an LSL stream and aligned with motion-capture and video data.

As part of the broader project [23], additional signals were also recorded:

- VR system logs: Positions of virtual hands and balls in the visuo-haptic VR environment.
- EEG: A 64-channel EEG (ActiveTwo; BioSemi B.V., Amsterdam, The Netherlands; 2048 Hz) on selected days.
- EMG: Wireless EMG (Trigno; Delsys Inc., Natick, MA, USA; 12 sensors) from upper-limb muscles on selected days.
- fMRI: Functional MRI scans before and after the entire training experiment.

Analyses of EEG, EMG, and fMRI are outside the scope of this study and will be reported separately.

#### 2.3.2. Real-World Three-Ball Juggling

In Sessions 1 and 4, participants performed real-world three-ball cascade juggling using LED glow balls under controlled lighting to improve tracking quality. Each daily session lasted about 5 min (15 min on Days 1, 5, and 10). Motion capture and RGB-D video were recorded continuously. These sessions were used to evaluate overall real-world juggling performance (Perf) and to provide the data for computing the Sequencing index (S).

#### 2.3.3. Visual-Occlusion Task

In Session 3, participants performed a two-ball juggling task while wearing liquid-crystal shutter glasses (PLATO Visual Occlusion Spectacles [25]). They alternately threw and caught two balls with opposite hands according to verbal instructions. An RGB-D camera recorded ball trajectories and hand movements, and a motion-capture system tracked markers placed on both shoulders and wrists.

Visual occlusion was controlled by an apex-locked system. Ball centroids were tracked online from a second RGB-D camera (Intel RealSense D435; Intel Corporation, Santa Clara, CA, USA; librealsense2 v2.49.0). The vertical position of each ball was temporally smoothed, and apex events were detected when the vertical velocity crossed zero while the vertical acceleration was negative. When the second ball reached its apex, both lenses of the shutter glasses were closed for 0.5 s, transiently blocking vision of the descending ball.

On most training days, participants completed 60 throw–catch repetitions. On Days 1, 5, and 10, the task consisted of four blocks of 75 attempts (300 trials in total), evenly divided among three conditions: (i) apex-locked occlusion, (ii) no occlusion, and (iii) randomly timed occlusion, with condition order randomized within each block. These trials provided the data to be used to compute the Prediction (P) and Accuracy (A) measures in the subsequent analyses.

### 2.4. Construction of Sequencing, Prediction, and Accuracy Indices

#### 2.4.1. Sequencing (S) from Real-World Three-Ball Juggling

**Figure 3:**
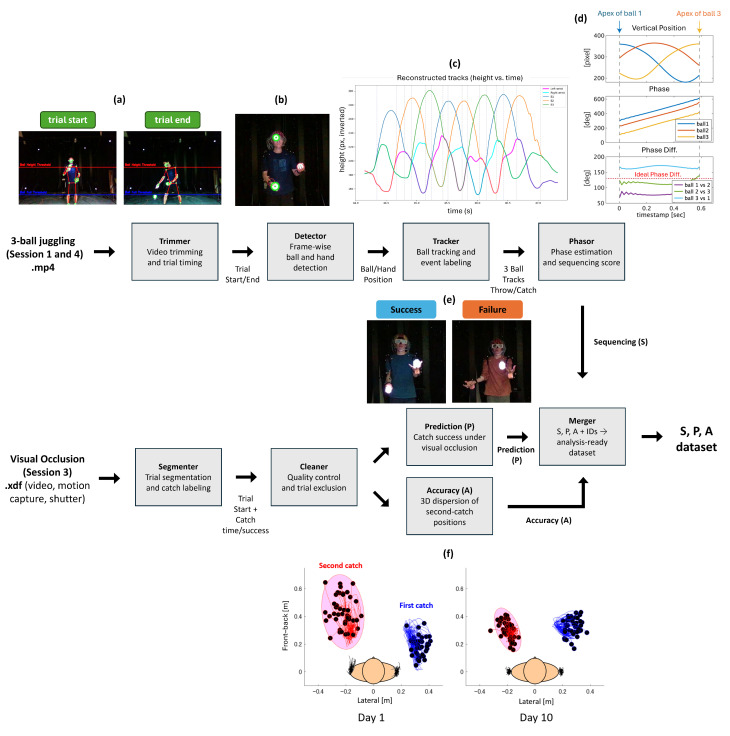
Overview of processing pipeline for constructing Sequencing, Prediction, and Accuracy. (**a**) Display of trimming the RGB video into proper trials. The red horizontal line is a shoulder threshold, while the blue line is a knee threshold. LED glow balls’ positions are estimated and marked as green circles. (**b**) Display of detecting balls and hands positions of each frame. The balls are labeled as green circles, while the hands are labeled as red circles. (**c**) A graphical example of reconstructed tracks of balls and hands by the tracker. The x-axis is time (s), and the y-axis is height (pixel). The blue, orange, and green lines describe trajectories of 3 different balls, while the pink and light blue lines show the vertical movements of both hands. Sequencing is computed based on these reconstructed balls’ phases. (**d**) Phase estimation in the Phasor stage. The top panel shows vertical trajectories of the three balls, with apex times used to define windows. The middle panel shows the instantaneous phase of each ball (degrees), and the bottom panel shows pairwise phase differences compared with the ideal 120° phase difference. (**e**) Example of success and failure of the visual-occlusion task for computing Prediction (P). (**f**) Illustration of probability ellipsoid for computing Accuracy (A). The x-axis is the lateral distance (m) from the center of a participant’s body, while the y-axis is the front–back distance (m) [29]. Each black dot connected with a blue line means the position of a first catch with the right hand (in this case), and each black dot connected with a red line means the position of a second catch with the left hand. Pink ovals represent the 95% probability ellipsoid of second catch positions of each day. Most participants show a decrease in the ellipsoid volume throughout the training period (left → right).
  To quantify the sequencing ability for each day, we pooled all valid sequencing windows ($S_{w}$) obtained from trials yielding at least one valid window across the analyzed sessions (Sessions 1 and 4). The day-level Sequencing index (*S*) was computed as the arithmetic mean of these pooled window values.

Sequencing (S) quantifies how well the three balls maintain a stable 120° phase relationship during real-world three-ball cascade juggling. To avoid extensive manual labeling, we designed an automatic Python (v3.12.7) pipeline to compute Sequencing with four stages (see Figure 3a–d): (i) video trimming and trial timing, (ii) frame-wise ball/hand detection, (iii) ball tracking and event labeling, and (iv) phase estimation and Sequencing score computation.

1. Video trimming and trial timing. The RGB video from Sessions 1 and 4 was segmented into individual trials using Python. Mediapipe Pose (v0.10.18) [26] was used to track both hands, and OpenCV (v4.12.0)-based blob detection [27] identified candidate ball positions. Based on the relative positions and movements of the hands and balls, the program automatically determined when each juggling trial began and ended (Figure 3a).
2. Frame-wise ball and hand detection. Within each trimmed juggling trial, videos were processed frame by frame in the second stage. For each frame, it recorded the detected ball candidates and the hand positions estimated by OpenCV [27] and Mediapipe [26]. These frame-wise detections were stored in a compact columnar format (parquet) and served as the input for the tracking stage (Figure 3b).
3. Ball tracking and event labeling. Using the frame-wise detections, the third stage reconstructed three continuous ball trajectories for each trial. Throws were labeled when a ball left the vicinity of a hand, and catches were labeled when a ball entered and stayed near the opposite hand. By connecting detections across frames, the algorithm followed each ball over time and produced smooth trajectories with associated throw and catch events (Figure 3c).
4. Phase estimation and Sequencing score. In the final stage, we used the reconstructed trajectories to compute phases and derive a Sequencing score (Figure 3d). For each ball b∈{1,2,3}, the smoothed and mean-subtracted vertical position ${\tilde{y}}_{b}\left(t\right)$ was converted to an instantaneous phase $\phi_{b}\left(t\right)$ using the analytic signal from the Hilbert transform [28],
  (1) $$a_{b}\left(t\right)={\tilde{y}}_{b}\left(t\right)+i\;H\left\{{\tilde{y}}_{b}\left(t\right)\right\},\;\phi_{b}\left(t\right)=\arg a_{b}\left(t\right),$$
  where H{·} denotes the Hilbert transform.
  For windows containing three consecutive apexes from three different balls, we quantified how closely the three phases maintained the ideal $120^{\circ }$ spacing. Let $\phi_{i}^{\circ }\left(t\right)$ be the phase of ball *i* in degrees and $t_{k}\in T_{w}$ the usable time samples within window *w* ($K=\;|T_{w}|$). The window-wise Sequencing score was defined as
  (2) $$S_{w}=\frac{1}{K}\sum_{k=1}^{K}\left[1-\frac{\delta_{12}\left(t_{k}\right)+\delta_{13}\left(t_{k}\right)+\delta_{23}\left(t_{k}\right)}{3\times 180^{\circ }}\right],$$
  where $\delta_{ij}\left(t_{k}\right)$ is the circular difference (modulo $360^{\circ }$) between $\phi_{j}^{\circ }\left(t_{k}\right)-\phi_{i}^{\circ }\left(t_{k}\right)$ and the ideal $120^{\circ }$ spacing. Thus, $S_{w}$ takes values in [0,1], with $S_{w}=1$ indicating perfectly maintained $120^{\circ }$ phase offsets.

#### 2.4.2. Prediction (P) and Accuracy (A) from the Visual-Occlusion Task

Prediction (P) and Accuracy (A) were derived from the visual-occlusion task in Session 3 using synchronized motion-capture and video recordings (Figure 3e,f).

1. Trial segmentation and labeling. We used Lab Streaming Layer (LSL) XDF files containing video timestamps, motion-capture markers for both wrists and shoulders, and shutter timing signals. In MATLAB (R2021b), continuous recordings were segmented into individual trials based on changes in hand velocity and acceleration, and each trial was saved as a separate .mat file. For each trial, we annotated both the timing and success of the catches for each hand (including the second catch). Trials with shutter malfunction, severe marker loss, or unclear outcomes were excluded from subsequent analyses.
2. Prediction (P): Catch success in the visual-occlusion task. Prediction (P) summarizes how reliably both hands complete the catches during the two-ball visual-occlusion task (Figure 3e). For each participant *p* and day *d*, let $N_{p,d}^{\mathrm{succ}}$ denote the number of valid trials in Session 3 in which both hands successfully completed the second catch, and let $N_{p,d}^{\mathrm{valid}}$ be the total number of valid trials on that day. The day-level Prediction index was defined as
  (3) $$P_{p,d}=\frac{N_{p,d}^{\mathrm{succ}}}{N_{p,d}^{\mathrm{valid}}},\;0\leq P_{p,d}\leq 1,$$
  with higher values indicating more reliable catch performance in the visual-occlusion task.
3. Accuracy (A): Spatial dispersion of catch locations. Accuracy (A) quantifies how tightly the second-catch positions cluster in 3D space (Figure 3f). This two-ball shutter-glasses paradigm was chosen because it has many controlled catch samples and allows us to quantify hand-centered spatial precision under reduced visual feedback, which is harder to measure reliably in short and unstable three-ball cascades during early learning. For each participant *p* and day *d*, we expressed the 3D position of each second catch in a hand-centered coordinate system defined by the corresponding wrist marker. In Session 3 on Days 1, 5, and 10, two-ball juggling was performed in four blocks; for each valid block $b\in B_{p,d}$ and each hand h∈{L,R}, all second-catch positions were pooled to form a 3D point cloud, and a 95% probability ellipsoid was fitted to this cloud, yielding an ellipsoid volume $V_{p,d,h,b}>0$.
  Day-level Accuracy was then defined as the geometric mean of these volumes,
  (4) $$A_{p,d}={\left(\prod_{h\in \{L,R\}}\prod_{b\in B_{p,d}}V_{p,d,h,b}\right)}^{1/\left(2|B_{p,d}|\right)},$$
  so that smaller values of $A_{p,d}$ indicate tighter clustering and, therefore, higher spatial Accuracy of the second catches. Blocks in which the ellipsoid fit was unstable (e.g., nearly singular covariance) were excluded before computing the geometric mean.
4. Aggregation and data merging. Day-level indices *P*, *A*, and *S* were summarized for Days 1, 5, and 10 and merged into a single dataset with participant and group information for the subsequent statistical analyses.

### 2.5. Statistical Modeling of Performance

#### 2.5.1. Data Preprocessing

All statistical analyses were conducted in MATLAB. From the preceding processing steps, we obtained a merged day-level dataset for each participant *p* and training day d∈{1,5,10}: (i) the three skill indices $S_{p,d}$, $P_{p,d}$, and $A_{p,d}$; (ii) a behavioral performance measure ${\mathrm{Perf}}_{p,d}$; and (iii) group labels (Low-g/Normal-g) and participant IDs. To ensure comparability and avoid bias from different practice amounts, the main analysis focused on three standardized test days (Days 1, 5, and 10), while the remaining days were used to describe trends and test robustness.

Day-level juggling performance ${\mathrm{Perf}}_{p,d}$ was defined from the real-world three-ball sessions (Sessions 1 and 4). For each participant and day, we pooled all trials from these two sessions, counted the number of successful catches in each trial, and identified the 10 trials with the longest successful catches. The performance measure ${\mathrm{Perf}}_{p,d}$ was then defined as the mean of successful catches across these top 10 trials.

Sequencing values $S_{p,d}$ were taken from the phase-based analysis described above. To ensure statistical reliability, we identified and excluded outliers based on the distribution of valid window counts ($N_{windows}$). Given the positively skewed nature of the data, we applied a log transformation and established a conservative lower bound of three standard deviations below the mean ($Mean_{log}-3SD_{log}$). This statistical analysis yielded a cutoff of approximately 4.5 windows. Accordingly, sessions falling below this threshold ($N_{windows}\leq 4$)—specifically, three participants on Day 1 who produced 0, 1, and 4 valid windows—were identified as statistical outliers. Since the Sequencing index is computed as a mean, such extremely low sample sizes inherently lead to inflated standard errors and unstable estimates. Therefore, these cases were set to missing for the day-level aggregation. We evaluated the robustness of excluding these cases in a sensitivity analysis reported in Section 3.3.

Since *S*, *P*, and *A* had different ranges and distribution shapes, we applied corresponding logit/log transforms and then z-transformation to them so that model coefficients reflect comparable effects.

Prediction values *P* are bounded between [0,1] and left-skewed, so we applied logit transform to it as follows:

(5) $$P_{p,d}^{\left(t\right)}=\log\;\left(\frac{{\tilde{P}}_{p,d}}{1-{\tilde{P}}_{p,d}}\right),\;{\tilde{P}}_{p,d}=\min\left(\max(P_{p,d},\varepsilon ),\;1-\varepsilon \right),\;\varepsilon =10^{-4}$$

Since Accuracy values *A* are positive with a long right tail, we applied a negative log transform so that larger *A* correspond to better Accuracy while stabilizing the distribution,

(6) $$A_{p,d}^{\left(t\right)}=-\log A_{p,d}$$

Sequencing values were used directly as follows: $S_{p,d}^{\left(t\right)}=S_{p,d}$.

For each transformed index $X^{\left(t\right)}\in \left\{P^{\left(t\right)},A^{\left(t\right)},S^{\left(t\right)}\right\}$, we then applied a robust *z*-transformation based on the median and median absolute deviation (MAD) to reduce sensitivity to outliers [30].

(7) $$Z_{X}=\frac{X^{\left(t\right)}-\mathrm{median}\left(X^{\left(t\right)}\right)}{1.4826\;\mathrm{MAD}(X^{\left(t\right)})}$$

**Figure 4:**
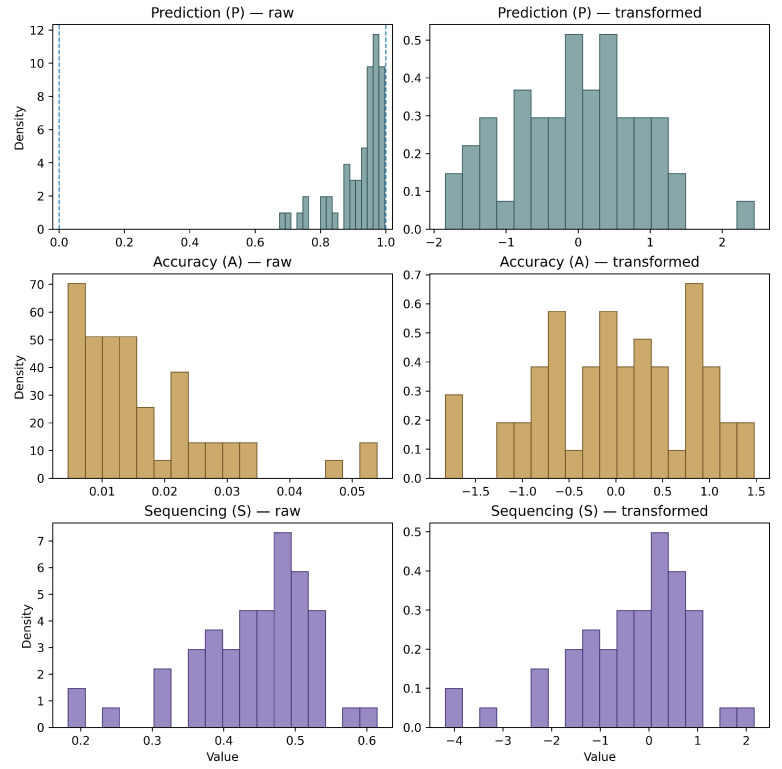
Distributions of raw and transformed skill indices. The left column shows pooled participant–day distributions of the raw indices: Prediction (*P*), Accuracy (*A*), and Sequencing (*S*). The right column shows the corresponding final indices after monotonic transforms (*P* is logit transformed, and *A* is negative log transformed) and robust z-transforms ($Z_{P}$, $Z_{A}$, and $Z_{S}$).

Figure 4 shows how preprocessing through monotonic transforms and z-transforms changed the distributions of the three indices. Raw Prediction (*P*) was bounded in (0,1) and highly concentrated near 1.0, showing a strong ceiling effect [31]. After the logit transform, the upper range was expanded, and the distribution became more symmetric and more suitable for linear modeling. Raw Accuracy (*A*) was strictly positive and right-skewed, with a small number of large-volume trials. The negative log transform reduced this skewness and reversed the direction, making the distribution more symmetric. Raw Sequencing (*S*) was already relatively well-behaved, so only the z-transform was applied to make it on the same standardized scale as *P* and *A*. Overall, the distinct raw distributions indicate that *S*, *P*, and *A* capture different behavioral properties rather than a single common factor, and the distributions of three skill metrics were improved by transforms to be more stable and suitable for fitting generalized linear models.

#### 2.5.2. Generalized Linear Models

Comparing with the Normal-identity generalized linear model (Normal-identity GLM) [32], this work presents a better fit using a Gamma-Log generalized linear model (Gamma-Log GLM) [33] to fit the pooled Day 1/5/10 data. In a Gamma-Log GLM, the expected performance $\mu_{p,d}=E\left[{\mathrm{Perf}}_{p,d}|Z_{P,p,d},Z_{A,p,d},Z_{S,p,d}\right]$ is linked to a linear predictor through the log function, while the response itself is assumed to follow a Gamma distribution:

(8) $${\mathrm{Perf}}_{p,d}∼\mathrm{Gamma}\left(\mu_{p,d},\;\phi \right),\;\log\left(\mu_{p,d}\right)=\beta_{0}+\beta_{P}Z_{P,p,d}+\beta_{A}Z_{A,p,d}+\beta_{S}Z_{S,p,d},$$

Gamma distribution is a better fit because the day-level juggling performance (Perf) is strictly positive and right-skewed, where there are many low values with a tail of high values. Compared to Normal distribution, Gamma distribution is specifically designed for positive continuous outcomes with skewness, while it also naturally captures the pattern where variability grows with the mean. With a log link, the model ensures positive predictions, while each skill index changes performance by a constant factor regardless of whether someone starts low or high.

#### 2.5.3. Model Evaluation and Comparison

In addition to reporting coefficient estimates and p-values, we evaluated model fitness using complementary fit criteria that are standard for GLMs and appropriate for our positive-valued performance outcome. Since the Gamma-Log GLM is estimated by maximum likelihood rather than least squares [33], likelihood-based indices (log-likelihood, Akaike information criterion (AIC) [34] and Cox–Snell pseudo-$R^{2}$ [35]) provide the most direct measures for illustrating goodness-of-fit for the model. To compare the predictive accuracy with Normal-Identity GLM, we also report the coefficient of determination ($R^{2}$) and Root Mean Square Error (RMSE), together with mean absolute error (MAE), which yields an interpretable error magnitude that is less sensitive to extreme outliers than RMSE [36]. Finally, given the large inter-individual variability in early motor learning, we used leave-one-subject-out cross-validation (LOSO-CV) [37] to test whether the fitted model generalizes to unseen participants. Together, these metrics show a comprehensive assessment of both in-sample fit and cross-subject generalization.

## 3. Results

### 3.1. Skill Metrics over Days

**Figure 5:**
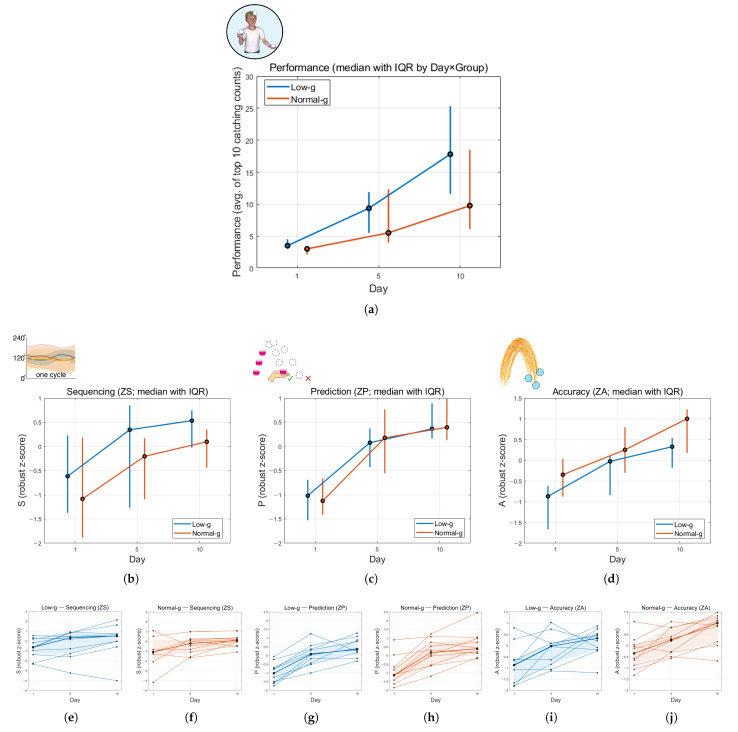
Day-wise medians and IQR (0.25–0.75) of Performance, Sequencing ($Z_{S}$), Prediction ($Z_{P}$), and Accuracy ($Z_{A}$). Low-g and Normal-g markers are horizontally offset slightly for better visibility, while both groups were actually measured on the same days. The blue and red lines represent the Low-g and Normal-g groups: (**a**) performance defined as the average of the top 10 catching counts; (**b**) Sequencing ($Z_{S}$) between different groups through days after robust z-transform; (**c**) Prediction ($Z_{P}$) between different groups through days after robust z-transform; (**d**) Accuracy ($Z_{A}$) between different groups through days after robust z-transform; (**e**–**j**) thin lines show individual trajectories of $Z_{S}$, $Z_{P}$, and $Z_{A}$ across Days 1, 5, and 10. Thick lines represent the group medians, and the shaded areas indicate the interquartile ranges (IQRs; 0.25–0.75). Sequencing is illustrated schematically by within-cycle phase relations. In the Prediction schematic, √/× denote successful/failed catch outcomes and dashed circles indicate example ball positions during visual occlusion. In the Accuracy schematic, circles indicate example catch locations.

Figure 5 and Table 1 summarize day-wise medians and interquartile ranges (IQR; 0.25–0.75) of juggling performance (Perf) (Figure 5a) and the three standardized skill indices ($Z_{S}$, $Z_{P}$, $Z_{A}$) (Figure 5b–d) for each group. We present medians with IQR because they provide a robust summary of central tendency and variability and are less sensitive to outliers. While all metrics show increases from Day 1 to Day 10, the improvement differences in each group are different. Although group differences were not statistically significant, the Low-g group showed a trend of higher median values in juggling performance (Perf) and Sequencing ($Z_{S}$) than the Normal-g group, while this trend was not observed in Prediction ($Z_{P}$) nor Accuracy ($Z_{A}$).

Juggling performance (Perf) increased in both groups through days, with a higher median trajectory in the Low-g group (Figure 5a). The Low-g group improved from Days 1 to 10 (median of 3.5 → 17.8), whereas the Normal-g group also improved but reached a lower median (median of 3.0 → 9.75). Sequencing ($Z_{S}$) also increased over time in both groups, and the Low-g group consistently showed higher median scores than the Normal-g group throughout the whole period (Figure 5b). In the Low-g group, $Z_{S}$ increased from −0.615 (Day 1) to 0.535 (Day 10), while in the Normal-g group, $Z_{S}$ increased from −1.08 (Day 1) to 0.098 (Day 10). Prediction ($Z_{P}$) also increased across days in both groups and showed very limited group difference by Day 10 (Figure 5c). Low-g changed from −1.02 (Day 1) to 0.364 (Day 10), while Normal-g changed from −1.13 (Day 1) to 0.395 (Day 10). Accuracy ($Z_{A}$) also increased over days in both groups (Figure 5d). However, the Normal-g group showed higher $Z_{A}$ than the Low-g group on all measured days. Low-g increased from −0.87 (Day 1) to 0.328 (Day 10), whereas Normal-g increased from −0.346 (Day 1) to 1.00 (Day 10).

While Low-g group showed higher Perf and Sequencing at all measured days, group differences were not significant (all *p* > 0.35), with small-to-moderate effect sizes. Considering the small sample size and limited training days, power was limited by the sample amount and high inter-individual variability.

Apart from group-median performance, each individual participant’s highly variable trajectories of $Z_{S}$, $Z_{P}$, and $Z_{A}$ across days are also presented in Figure 5e–j. Although the median robust z-scores increased from Days 1 to 10 in both groups, participants were highly different in baseline level and rate of change. Several individuals showed non-monotonic trajectories, including decreases across days, even within the Low-g group. This heterogeneity indicates that early juggling learning is strongly participant-specific, with a small sample number n=10 per group, a small number of fluctuating performers can substantially influence group-level summaries.

Before fitting the generalized linear models, we examined the relationships among the three standardized indices ($Z_{P}$, $Z_{A}$, $Z_{S}$). Pairwise Pearson correlations [38] were $r(Z_{P},Z_{A})=0.579$ ($p=2.37\times 10^{-6}$), $r(Z_{P},Z_{S})=0.329$ (p=0.012), and $r(Z_{A},Z_{S})=0.185$ (p=0.168). To assess collinearity in the regression context, we also computed variance inflation factors (VIFs) [39] from an ordinary least squares model with Perf as the outcome; VIF values were low (VI$F_{Z_{P}}=1.63$, VI$F_{Z_{A}}=1.51$, VI$F_{Z_{S}}=1.12$), which is well below common thresholds for collinearity (e.g., 5–10), indicating no problematic multicollinearity among the three predictors.

### 3.2. Results of Computational Modeling

Table 2 reports the Gamma-Log GLM coefficients estimated from standardized predictors $\left(Z_{P},Z_{A},Z_{S}\right)$. All coefficients are positive, indicating that higher Prediction, Accuracy, and Sequencing are associated with better juggling performance. Sequencing shows the strongest effect and is highly significant ($\beta_{S}=0.5279$, SE = 0.1036, p<0.001), followed by Prediction ($\beta_{P}=0.4073$, SE = 0.1766, p=0.021). Accuracy has a smaller coefficient and is not significant in the model ($\beta_{A}=0.1669$, SE = 0.1838, p=0.364), with a 95% CI that includes zero. Because the model uses a log link, exp(β) gives the multiplicative change in expected performance in each predictor: $\exp(\beta_{S})=1.695$ and $\exp(\beta_{P})=1.503$ correspond to about 70% and 50% increases in expected performance, respectively, while $\exp(\beta_{A})=1.182$ corresponds to an 18% increase but without statistical support.

We compared two generalized linear models using the standardized skill indices $\left(Z_{P},Z_{A},Z_{S}\right)$ pooled across Days 1, 5, and 10: (i) a Gamma GLM with a log link (Gamma-Log) and (ii) a Normal GLM with an identity link (Normal-Identity). Table 3 shows that the Gamma-Log model performed better across all evaluation criteria. Gamma-Log achieved higher explanatory power and lower prediction error ($R^{2}=0.427$, RMSE =25.77, MAE =11.40) than Normal-Identity ($R^{2}=0.277$, RMSE =28.95, MAE =18.04). Likelihood-based evidence also supported Gamma-Log GLM, with a higher log-likelihood (−188.63 vs. −272.71), lower AIC (385.26 vs. 553.43), and higher Cox–Snell pseudo-$R^{2}$ (0.701 vs. 0.301). Leave-one-subject-out cross-validation confirmed better generalization of Gamma–Log (LOSO $R^{2}=0.270$, RMSE =29.08, MAE =12.49) compared with Normal-Identity (LOSO $R^{2}=0.039$, RMSE =33.36, MAE =20.74). These results illustrate that Gamma-log GLM is a better fit as the computational model of juggling performance.

Table 4 reports fit metrics computed separately for each day. On Day 1, both models report $R^{2}=0.000$, indicating no explained variance at that early stage; still, the Gamma-Log model shows a smaller RMSE than the Normal-Identity model (5.17 vs. 15.10). On Day 5, the Gamma-Log model explains more variance ($R^{2}=0.168$) and has lower error (RMSE = 17.90) than the Normal-Identity model ($R^{2}=0.035$, RMSE = 19.28). On Day 10, both models improve, with Gamma-Log again higher in $R^{2}$ (0.375 vs. 0.265) and lower in RMSE (39.36 vs. 42.69). Across days, $R^{2}$ increases from Days 1 to 10 for both models, while RMSE also increases, reflecting larger absolute performance values at later days. Overall, the Gamma-Log model provides better day-wise fit than the Normal-Identity model on Days 5 and 10.

**Figure 6:**
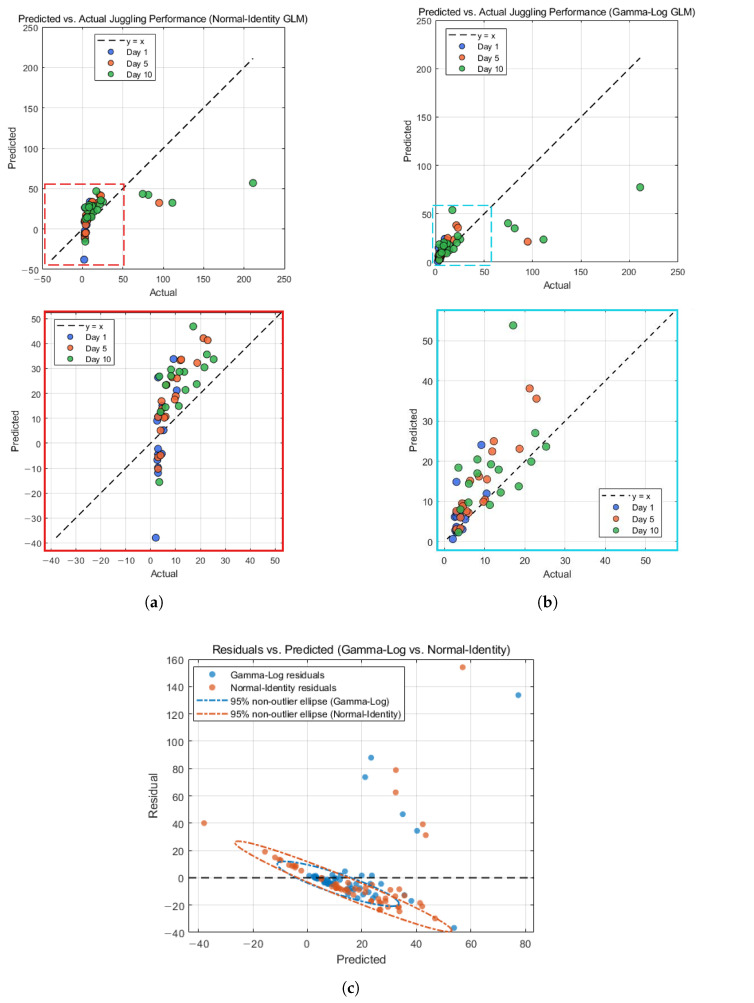
Predicted vs. observed juggling performance on Days 1, 5, and 10. The x- and y-axes represent the observed and predicted juggling performance from the models. Blue, orange, and green dots indicate individual juggling performance on Days 1, 5, and 10, respectively. Proximity between a dot and a dashed line (y = x) indicates how well the model can predict the juggling performance of the Participant–Day samples. (**a**) Normal-Identity GLM; (**b**) Gamma-Log GLM; (**c**) residuals vs. predicted juggling performance for the two GLMs. Blue dots indicate Participant–Day samples from the Gamma-Log GLM, and orange dots indicate samples from the Normal-Identity GLM. The x-axis shows each model’s predicted performance, and the y-axis shows the residual (observed − predicted Perf); the horizontal black (dashed) reference line marks zero residual. Dashed ellipses depict the 95% probability region of residuals within the non-outlier performance range.

Figure 6 summarizes the fit of the Gamma-Log model, with the Normal-Identity model shown as a reference check. Figure 6a shows the predicted versus observed performance for the Normal-Identity GLM. The wide spread around the identity line, especially at higher Perf, indicates limited fit for this one-sided outcome. Figure 6b shows the same comparison for the Gamma-Log GLM. Predictions follow the identity line more closely within the dense low-to-mid performance region, which covers most observations, although several high-performance points still deviate from the model. Figure 6c plots residuals against predicted performance for both models. The non-outlier range was defined on observed Perf by excluding MAD-based upper-tail outliers, i.e., trials with Perf>median(Perf)+3×(1.4826·MAD) (cutoff =19.79). Within this non-outlier range, Gamma-Log residuals show smaller robust bias and absolute deviation than the Normal model (Gamma-Log: med(res)=−3.57, med(|res|)=3.75; Normal: med(res)=−7.78, med(|res|)=9.43), consistent with the tighter, more zero-centered non-outlier ellipse in Sub-figure (c). Both models still show large positive residuals for a small number of high-performance cases, reflecting underestimation in the upper tail. Overall, the figure supports using the Gamma-Log GLM as a suitable computational description of early juggling performance, consistent with the pooled and by-day fit metrics in Table 3 and Table 4.

### 3.3. Sensitivity Analyses

As an exploratory extension of the main model, we added interaction terms between the standardized indices, as shown in Table 5. A Gamma-Log GLM including $Z_{P}$, $Z_{A}$, $Z_{S}$, and their pairwise interactions ($Z_{P}\;:\;Z_{A}$, $Z_{P}\;:\;Z_{S}$, $Z_{A}\;:\;Z_{S}$), improved overall fit relative to the base model (AIC = 382.96 vs. 385.26; $R^{2}=0.647$, RMSE = 20.21), while preserving the pattern that Sequencing and Prediction had positive coefficients and Accuracy was not significant. When we further included Group and Day as fixed effects together with the interaction terms, model fitness increased again (AIC = 382.25; $R^{2}=0.709$, RMSE = 18.37). In this extended model, $Z_{S}$ remained a robust positive variable, $Z_{P}$ showed a positive trend (p=0.058), and the $Z_{P}\;:\;Z_{S}$ interaction was significant (p=0.041), suggesting that the effect of Prediction is stronger when Sequencing is high. Other interactions were not significant.

To partially account for participant-specific baselines, we also fitted an extended GLM that added fixed intercepts for each participant with the skill interactions, Group, and Day. This model achieved the best overall fit (AIC = 303.31; $R^{2}=0.91$, RMSE = 10.18). Sequencing still remained a significant positive predictor ($\beta_{Z_{S}}=0.22$, p=0.013), while the main-effect coefficients for Prediction and Accuracy became slightly negative ($\beta_{Z_{P}}=-0.27$, p=0.067; $\beta_{Z_{A}}=-0.07$, p=0.60) despite significant positive interactions with Sequencing. Given the small sample size (n=20) and large number of parameters in this model, we interpret these sign changes cautiously as likely reflecting overfitting and partialing among correlated terms. These sensitivity analyses support the robustness of the main Gamma-Log results: Sequencing shows the most stable association with performance, Prediction contributes in interaction with Sequencing, and Accuracy has only a weak independent effect in this dataset.

Finally, to assess whether excluding the three Day 1 statistical outliers ($N_{windows}\leq 4$) introduced selection bias, we refitted the base Gamma-Log GLM on the full dataset (n=60). For this robustness check, we included the actual calculated (but unstable) $Z_{S}$ values for the two participants with one and four windows, and imputed the standardized median ($Z_{S}=0$) for the single participant with no valid windows. Coefficients for the core predictors changed only minimally (all shifts remained well within their respective standard errors) relative to the complete-case fit (n=57): $\Delta \beta_{Z_{P}}=+0.0343$, $\Delta \beta_{Z_{A}}=+0.0068$, and $\Delta \beta_{Z_{S}}=-0.0012$. Consequently, the qualitative conclusions were unchanged (Sequencing and Prediction: positive; Accuracy: non-significant). Fit indices were also comparable (complete case: AIC = 385.26, RMSE = 25.77; full-dataset imputation: AIC = 396.99, RMSE = 25.39).

## 4. Discussion

This study built a component-based computational model for early juggling learning using three sub-skills: Sequencing (S), Prediction (P), and Accuracy (A). With pooled data from Days 1, 5, and 10, we explored our three hypotheses about how these components form juggling skill and relate to juggling performance. The main findings are as follows:

1. H1. The three indices showed different distributions and behaviors, supporting the idea that early juggling skill can be separated into Sequencing, Prediction, and Accuracy (Figure 4).
2. H2. Day-level juggling performance can be explained by the three components together. All components had positive effects on juggling proficiency Perf, and Sequencing and Prediction showed the strongest independent contributions to it in the model (Table 2).
3. H3. The sub-skills did not increase at the same rate across days, showing lead–lag development. In line with our expectation, Sequencing rose steadily and remained higher in the Low-g group, suggesting that slow-tempo low-gravity VR training mainly supported early Sequencing gains (Figure 5).
4. Beyond these hypotheses, we observed large individual differences in learning trajectories, and a few very high-performance points were not well captured by the model (Figure 6).

### 4.1. Sub-Skill Contributions to Real-World Juggling Performance

In the Gamma-Log model, all three sub-skills had positive coefficients, meaning that higher S, P, and A were all linked to higher real-world juggling performance. Among them, Sequencing had the largest effect and was strongly significant (Table 2). This suggests that early performance mainly depends on building stable three-ball coordination over time, captured by rhythmic and phase-based measures. This agrees with the general idea that early jugglers first need to form a reliable cascade rhythm before they can sustain long runs.

Prediction was also significant. This suggests that the Prediction index, measured from apex-occlusion trials, captures a more general ability to estimate ball flight and landing without relying on continuous visual feedback. Participants who showed more reliable catching under occlusion tended to have higher three-ball performance even after controlling for S and A. Therefore, Prediction appears to be a real and measurable part of juggling skill that supports real-world performance rather than a task-specific artifact of the occlusion session.

Accuracy had a positive coefficient but was not significant in the computational model. This means that better spatial consistency of catch locations was related to higher Perf, but its unique contribution was smaller than Sequencing and Prediction at this early stage. We interpret this primarily as a limitation of the current metric rather than evidence that spatial accuracy is unimportant for juggling. By design, *A* is estimated from second-catch clustering in a two-ball shutter-glasses task, which provides many controlled samples and isolates hand-centered spatial precision under reduced visual feedback, but does not capture all spatial demands of three-ball cascade juggling (e.g., full height and azimuth variability over long sequences). In the present dataset, the number of stable three-ball cycles with reliable 3D catch positions was too limited and heterogeneous across days to support a robust, parallel kinematic variability index, so a formal validation of *A* against three-ball dispersion was not feasible. Therefore, we treat *A* as a conservative occlusion-based proxy for spatial control in early learning.

Although Prediction and Accuracy were both derived from the visual-occlusion session, they did not collapse onto a single measure. Prediction ($Z_{P}$) and Accuracy ($Z_{A}$) showed a moderate correlation (r=0.579, $p=2.37\times 10^{-6}$), but multicollinearity diagnostics remained low (all VIF <1.7), suggesting that the two indices contribute separable information when modeled jointly. This aligns with their operational definitions: Prediction reflects the probability of successful catches under occlusion, whereas Accuracy captures the spatial clustering of second-catch locations.

As stated in our research Hypothesis H1, juggling skill can be decomposed into three components. The different distributions of S, P, and A and their positive contributions support this hypothesis, and that performance can be modeled as their joint function (Hypothesis H2).

### 4.2. Learning-Stage Dependence and Asymmetric Development

Day-wise model evaluation showed that fitness increased from Days 1 to 10 (Table 4). Both models explained almost no variance on Day 1, while fitness improved on Day 5 and further on Day 10, indicating that the three sub-skill indices relate to real-world performance more clearly as learners build a stable cascade. On Day 1, most trials were short and unstable, and some indices were close to baseline or missing, especially Sequencing when too few valid phase windows were available. With practice, the indices expanded in range and tracked Perf more consistently.

The three components also changed at different rates across days. Sequencing increased steadily and remained higher in the Low-g group, suggesting that slow-tempo low-gravity VR training may preferentially support early Sequencing gains. Prediction improved strongly in both groups and became similar by Day 10, while Accuracy showed a different pattern and stayed higher in the Normal-g group (Figure 5). Given the modest sample size, these group patterns should be interpreted as trends rather than definitive group effects. These non-parallel trajectories support the lead–lag idea in H3, showing that early juggling learning involves multiple sub-skills that develop at different speeds, rather than a single skill improving uniformly.

### 4.3. Inter-Individual Variability and High-Performance Tail

Another clear outcome was strong variability between participants. Individuals differed in starting level and learning slope, and some showed non-monotonic changes. Baseline differences between participants were already large on Day 1, even though they were all beginners. Performance also fluctuated across days, which is common in motor learning, for example, due to fatigue, attention changes, or health conditions. With a small sample size (only n=10) per group and only three modeled days, these individual differences can largely influence group summaries, limit day-specific fit, and lower cross-subject prediction. The LOSO-CV result for Gamma-Log ($R^{2}=0.270$) shows that the model can generalize to new participants, but also that a large part of performance variance is still person-specific.

Although the Gamma-Log GLM provided the appropriate description of the data overall, the residual plots (Figure 6c) still show systematic misfit for a few high-performance observations. One likely reason is the sparse upper tail of the performance distribution: only a small number of participants achieved very long cascades, so the GLM is dominated by the dense low-to-moderate range and shrinks extreme values toward the central trend. In addition, there may be practical ceiling effects due to the finite trial duration and experimental constraints. Finally, high performers may rely on qualitatively different control strategies (e.g., more automated rhythm control or different gaze patterns), which are not well captured by a single global log-linear relationship between $\left(Z_{S},Z_{P},Z_{A}\right)$ and performance. Future work could explore alternative GLM families that allow more flexible variance structures, such as Tweedie [40] or inverse Gaussian distributions [32], but the current sample size is limited for reliably estimating and comparing such larger models. Therefore, we interpret the Gamma-Log model as a good description of typical early-stage performance while acknowledging reduced accuracy in the extreme high-performance tail.

### 4.4. Computational Model and Goodness-of-Fit

We used a Gamma-Log GLM as the main computational model because the performance outcome is strictly positive and right-skewed in early juggling. In this case, a Gamma distribution with a log link matches the scale of the data and naturally allows variance to increase with higher performance. The results showed that the Gamma-Log model captured the non-outlier performance range well and generalized reasonably across subjects in LOSO-CV (Table 3).

We also fitted a Normal-Identity GLM as a reference check. As expected for a one-sided outcome, its fit was weaker overall, and residual patterns were more biased than those of Gamma-Log (Figure 6). This study’s choice of the Gamma-Log model provides an appropriate statistical description of positive and heteroscedastic juggling performance, and this approach can be extended to other motor learning studies with similar outcome distributions.

Nonetheless, sensitivity analysis with pairwise interaction terms (Table 5) indicated a significant $Z_{P}\;:\;Z_{S}$ interaction when Group and Day were included, implying that the benefit of Prediction may be higher when Sequencing is already stable. Importantly, these extended models did not change the qualitative pattern of the main results: Sequencing and Prediction remained positive predictors of performance, whereas Accuracy showed only a small, non-significant contribution.

Because observations from the same participant were collected on multiple days, we also fitted an extended Gamma-Log GLM that included fixed intercepts for each participant in addition to Group, Day, and the skill interactions. In this model, Sequencing remained a significant positive predictor, while the main-effect coefficients for Prediction and Accuracy became slightly negative, despite positive interactions with Sequencing. Given the small sample size (n=20) and the large number of parameters, we interpret these sign changes cautiously as likely reflecting overfitting and partialing among correlated terms, rather than a reversal of the underlying relationships. For this reason, we retain the simple Gamma-Log GLM as the primary model and use the interaction effects and participant-intercept models only as complementary sensitivity checks.

In addition to these model-form robustness checks, we also examined whether excluding the Day 1 statistical outliers ($N_{windows}\leq 4$) introduced selection bias. We refitted the base Gamma-Log GLM on the full dataset (n=60) by retaining the actual $Z_{S}$ values for the cases with 1 and 4 windows despite their instability, and imputing the standardized median ($Z_{S}=0$) for the case with no valid windows. This produced only minimal changes in the core coefficients ($\Delta \beta_{Z_{P}}=+0.0343$, $\Delta \beta_{Z_{A}}=+0.0068$, $\Delta \beta_{Z_{S}}=-0.0012$) and similar fit indices, indicating that the main inferences are not driven by the Day 1 exclusions.

### 4.5. Limitations

This study has several limitations. First, the sample size (n=20) was relatively small for evaluating the training intervention. Although the Low-g group exhibited a trend of higher performance and Sequencing scores, the observed effect sizes were small to moderate (Hedges’ g ≈ 0.26–0.40 for Perf). A post hoc power analysis indicated that a sample size of over 100 participants (approx. 139) would be required to definitively confirm the efficacy of Low-g training with sufficient statistical power. However, as the primary focus of this study was to decompose juggling skills into computational components rather than to validate the training effect itself, we interpret the group-level trends descriptively.

Second, the main Gamma-Log GLM treated Participant–Day samples as independent, although each participant contributed multiple days. This may induce correlated residuals; an exploratory mixed-effects model with participant-level random intercepts showed similar patterns but was not adopted as the primary model, and future work with larger samples should use mixed-effects Gamma models more systematically. Second, both tested GLMs were additive and still underestimated a few very-high-performance cases, suggesting that models with nonlinear or interaction terms, or alternative positive distributions, could better capture the upper tail. Third, Accuracy was measured only by ellipsoid volume from the two-ball occlusion task. Spatial accuracy in three-ball juggling also includes throw height and direction consistency, which may become more important later.

### 4.6. Future Work

Future studies should extend the model to all training days and increase the sample size so that more complex structures can be estimated reliably. In particular, mixed-effects Gamma models with participant-specific random intercepts could more reliably model the repeated-measures structure directly and test whether component weights change across learning stages (e.g., Day $\times \;Z_{S}$, Day $\times \;Z_{P}$, Day $\times \;Z_{A}$) with sufficient data. In addition, the underestimation of very high performers suggests that nonlinear terms or interaction effects among the components (e.g., $Z_{S}\times Z_{P}$) may capture residual variance that the current simple GLM cannot explain.

The current Accuracy index captures only part of the spatial precision required in full three-ball cascade juggling because it is based on two-ball occlusion catches. Future work should derive complementary Accuracy measures directly from three-ball kinematics (e.g., variability of catch height and coordinates, horizontal error distributions) and test how these measures relate to Perf and to the present two-ball-based index *A*. This will allow a more complete assessment of spatial control as a component of juggling skill.

Beyond the behavioral and kinematic results presented here, the 10-day multimodal evaluation system also included EEG, EMG, and pre-/post-fMRI recordings [23]. In future work, these modalities could be integrated with the three-component model to relate Sequencing, Prediction, and Accuracy to patterns of brain activity, functional connectivity, and muscle activation. For example, EEG and fMRI could be used to examine how training-related changes in cortical dynamics covary with improvements in each sub-skill, and whether low-gravity VR training preferentially modifies networks related to temporal coordination, while EMG could quantify how motor efficiency and muscle usage evolve with practice. This multimodal extension would provide a more complete picture of how complex motor skills such as juggling are acquired through training in both brain and body.

Although the present work focuses on three-ball cascade juggling, the proposed three-component structure is likely relevant for other complex motor skills. In racket and ball sports (e.g., tennis, table tennis, basketball), athletes must learn stable action sequences and rhythms (Sequencing), predict ball trajectories and player movements under uncertainty (Prediction), and place the racket, hand, or foot at precise spatial targets (Accuracy). Similarly, in musical performance (e.g., piano and drumming), learners acquire ordered finger and limb patterns (Sequencing), anticipate upcoming notes and phrases (Prediction), and control spatial endpoints such as key locations or hand positions (Accuracy). These examples suggest that a Sequencing–Prediction–Accuracy decomposition may provide a general template for analyzing how multiple sub-skills support proficiency in real-world motor tasks beyond juggling. Our results show that these elements can be quantified separately and may develop at different speeds in early learning. Thus, the present framework may provide a general starting point for decomposing and modeling other complex motor skills in future work.

## 5. Conclusions

This study introduces a longitudinal learning study focusing on the behavioral and kinematic results from our larger multimodal juggling project. In conclusion, our computational model using Gamma-Log GLM shows that early juggling performance can be described as a combined function of Sequencing, Prediction, and Accuracy. Among them, Sequencing has the strongest positive associations with juggling performance, followed by Prediction, and then Accuracy. Across days, the three components followed different learning trajectories, and Sequencing increased most clearly in the low-gravity group, consistent with the expectation that slow-tempo low-gravity VR practice may potentially promote early rhythmic coordination. Overall, this study provides a structured, multi-component view of early juggling acquisition and offers a clear computational framework for interpreting motor learning in complex real-world tasks.

## Figures and Tables

**Table 1:** Day-by-Group summary of Performance, Sequencing ($Z_{S}$), Prediction ($Z_{P}$), and Accuracy ($Z_{A}$), as shown by median with [IQR].

Day	Group	Perf	$Z_{S}$ (Sequencing)	$Z_{P}$ (Prediction)	$Z_{A}$ (Accuracy)
1	Low-g	3.5 [3, 4.5]	−0.615 [−1.37, 0.228]	−1.02 [−1.53, −0.69]	−0.87 [−1.66, −0.616]
1	Normal-g	3 [2.1, 3]	−1.08 [−1.88, 0.185]	−1.13 [−1.41, −0.659]	−0.346 [−0.877, 0.0361]
5	Low-g	9.35 [5.5, 11.9]	0.346 [−1.27, 0.852]	0.0778 [−0.432, 0.374]	−0.0247 [−0.839, 0.104]
5	Normal-g	5.5 [4, 12.3]	−0.203 [−1.09, 0.173]	0.178 [−0.556, 0.766]	0.254 [−0.303, 0.797]
10	Low-g	17.8 [11.6, 25.3]	0.535 [−0.0257, 0.75]	0.364 [0.164, 0.892]	0.328 [−0.178, 0.54]
10	Normal-g	9.75 [6.1, 18.5]	0.0981 [−0.438, 0.354]	0.395 [0.133, 0.983]	1 [0.175, 1.23]

**Table 2:** Results of the Gamma-Log GLM for juggling performance; coefficient estimates for $\left(Z_{P},Z_{A},Z_{S}\right)$ are shown with standard errors (SEs), Wald *z* statistics, *p* values, 95% confidence intervals, and multiplicative effects exp(β).

Predictor	β	SE	*z*	*p*	95% CI	exp(β)
Intercept	2.5563	0.1276	20.043	<0.001 ***	[2.306, 2.806]	–
$Z_{P}$	0.4073	0.1766	2.307	0.021 *	[0.061, 0.753]	1.503
$Z_{A}$	0.1669	0.1838	0.908	0.364	[−0.193, 0.527]	1.182
$Z_{S}$	0.5279	0.1036	5.092	<0.001 ***	[0.325, 0.731]	1.695

Notes. Significance codes: * p<0.05, *** p<0.001.

**Table 3:** Results of model evaluation for juggling performance; two generalized linear models (Gamma-Log and Normal-Identity) were fitted using $\left(Z_{P},Z_{A},Z_{S}\right)$ across Days 1/5/10, and their fit and prediction accuracy are reported by log-likelihood, AIC, Cox–Snell pseudo-$R^{2}$, pooled $R^{2}$/RMSE/MAE, and leave-one-subject-out cross-validation (LOSO-CV) $R^{2}$/RMSE/MAE.

Model	LogLik	AIC	Pseudo-$R^{2}$ (CS)	$R^{2}$/RMSE/MAE	LOSO $R^{2}$/RMSE/MAE
Gamma–Log	−188.63	385.26	0.701	0.427 / 25.77 / 11.40	0.270 / 29.08 / 12.49
Normal–Identity	−272.71	553.43	0.301	0.277/28.95/18.04	0.039/33.36/20.74

**Table 4:** Results of day-wise model evaluation; $R^{2}$ and RMSE are reported separately for Days 1, 5, and 10 for Gamma-Log and Normal-Identity GLMs.

	Gamma-Log		Normal-Identity	
Day	$R^{2}$	RMSE	$R^{2}$	RMSE
Day 1	0.000	5.17	0.000	15.10
Day 5	0.168	17.90	0.035	19.28
Day 10	0.375	39.36	0.265	42.69

**Table 5:** Sensitivity analyses extending the main Gamma-Log model. Fixed-effect coefficients are shown for the standardized skill indices, together with key interaction terms and overall fit indices.

Model	$\beta_{Z_{P}}$	$\beta_{Z_{A}}$	$\beta_{Z_{S}}$	Key Interaction	AIC	$R^{2}$/RMSE
Gamma-Log (base)	0.41 *	0.17	0.53 ***	–	385.26	0.43/25.77
Base + full dataset ($Z_{S}$ imputed)	0.44 *	0.17	0.53 ***	–	396.99	0.42/25.39
Base + skill interactions	0.54 **	0.06	0.55 ***	$Z_{P}\;:\;Z_{S}=0.14$	382.96	0.65/20.21
Base + skill interactions + Group + Day	0.39 †	0.01	0.50 ***	$Z_{P}\;:\;Z_{S}=0.19$ *	382.25	0.71/18.37
Base + interactions + Group + Day + subject	$-0.27^{\;†}$	−0.07	0.22 *	$Z_{P}\;:\;Z_{S}=0.13$ * $Z_{A}\;:\;Z_{S}=0.24$ **	303.31	0.91/10.18

Notes. Significance codes: ^†^  p<0.10, * p<0.05, ** p<0.01, *** p<0.001.

## Data Availability

The data presented in this study are not publicly available due to ethical and privacy restrictions. The dataset includes human participant recordings and also contains ongoing, not-yet-published data. Anonymized data may be made available from the corresponding author upon reasonable request and with approval from the responsible ethics committee.

## Abbreviations

The following abbreviations are used in this manuscript:

VR	Virtual reality
GLM	Generalized linear model
DTI	Diffusion tensor imaging
VBM	Voxel-based morphometry
PCA	Principal component analysis
FDA	Functional data analysis
N-T-C	Noise reduction, tolerance exploitation, and covariation
EEG	Electroencephalography
EMG	Electromyography
*S*	Sequencing (index)
*P*	Prediction (index)
*A*	Accuracy (index)
fMRI	Functional magnetic resonance imaging
Perf	Performance (juggling performance)
LSL	Lab streaming layer
LED	Light-emitting diode
MAD	Median absolute deviation
AIC	Akaike information criterion
$R^{2}$	Coefficient of determination
RMSE	Root mean squared error
MAE	Mean absolute error
LOSO-CV	Leave-one-subject-out cross-validation
VIF	Variance inflation factor
IQR	Interquartile range
CI	Confidence interval
SE	Standard error
LogLik	Log-likelihood
